# BSG (CD147) Serum Level and Genetic Variants Are Associated with Overall Survival in Acute Myeloid Leukaemia

**DOI:** 10.3390/jcm11020332

**Published:** 2022-01-10

**Authors:** Piotr Łacina, Aleksandra Butrym, Eliza Turlej, Martyna Stachowicz-Suhs, Joanna Wietrzyk, Grzegorz Mazur, Katarzyna Bogunia-Kubik

**Affiliations:** 1 Laboratory of Clinical Immunogenetics and Pharmacogenetics, Hirszfeld Institute of Immunology and Experimental Therapy, Polish Academy of Sciences, 53-114 Wroclaw, Poland; katarzyna.bogunia-kubik@hirszfeld.pl; 2 Department of Cancer Prevention and Therapy, Wroclaw Medical University, 50-556 Wroclaw, Poland; aleksandra.butrym@umw.edu.pl; 3 Department of Experimental Oncology, Hirszfeld Institute of Immunology and Experimental Therapy, Polish Academy of Sciences, 53-114 Wroclaw, Poland; eliza.turlej@upwr.edu.pl (E.T.); martyna.stachowicz@hirszfeld.pl (M.S.-S.); joanna.wietrzyk@hirszfeld.pl (J.W.); 4 Department of Experimental Biology, Wrocław University of Environmental and Life Sciences, 50-375 Wroclaw, Poland; 5 Department of Internal, Occupational Diseases, Hypertension and Clinical Oncology, Wroclaw Medical University, 50-556 Wroclaw, Poland; grzegorz.mazur@umw.edu.pl

**Keywords:** basigin, CD147, monocarboxylate transporter 1, acute myeloid leukemia, single nucleotide polymorphism, survival

## Abstract

Basigin (BSG, CD147) is a multifunctional protein involved in cancer cell survival, mostly by controlling lactate transport through its interaction with monocarboxylate transporters (MCTs) such as MCT1. Previous studies have found that single nucleotide polymorphisms (SNPs) in the gene coding for BSG and MCT1, as well as levels of the soluble form of BSG (sBSG), are potential biomarkers in various diseases. The goal of this study was to confirm BSG and MCT1 RNA overexpression in AML cell lines, as well as to analyse soluble BSG levels and selected BSG/MCT1 genetic variants as potential biomarkers in AML patients. We found that BSG and MCT1 were overexpressed in most AML cell lines. Soluble BSG was increased in AML patients compared to healthy controls, and correlated with various clinical parameters. High soluble BSG was associated with worse overall survival, higher bone marrow blast percentage, and higher white blood cell count. BSG SNPs rs4919859 and rs4682, as well as MCT1 SNP rs1049434, were also associated with overall survival of AML patients. In conclusion, this study confirms the importance of BSG/MCT1 in AML, and suggests that soluble BSG and BSG/MCT1 genetic variants may act as potential AML biomarkers.

## 1. Introduction

Acute myeloid leukaemia (AML) is a haematologic malignancy associated with uncontrolled proliferation of immature leukemic blasts that can lead to bone marrow failure [1]. It is the most commonly diagnosed acute leukaemia in adults and its prognosis remains poor, with the 5-year overall survival rate being only 25% [2]. While various prognostic markers exist, the highly heterogeneous nature of AML means that treatment outcomes can vary widely between patients [3]. Therefore, new diagnostic and prognostic markers are needed.

Basigin (BSG), also known as cluster of differentiation 147 (CD147) and extracellular matrix metalloproteinase inducer (EMMPRIN), is a glycoprotein that belongs to the immunoglobulin superfamily. It is a transmembrane protein commonly expressed on many human cell types and first identified as a stimulator of matrix metalloproteinase (MMP) production, although it is now known to also be involved in many other cellular pathways [4,5]. It was recently found to be an alternative entry receptor for the SARS-CoV-2 virus, contributing to its spread [6,7]. BSG expression is increased in many cancers, and it is considered to be a biomarker for cancer diagnosis and prognosis [8,9,10]. It is known to promote tumour invasiveness and metastasis by stimulating MMP production [11]. However, it was shown that its pro-tumour function is mostly due to its interaction with monocarboxylate transporters (MCTs), such as MCT1 (also known as SLC16A1) and MCT4 (also known as SLC16A3), rather than MMPs [12]. MCTs are membrane-bound proteins that mediate transport of lactic acid across the membrane [13]. MCTs are especially important in cells using glycolysis for energy generation, as increased lactate production through glycolysis can drastically decrease cytosolic pH and lead to cell death through acidosis [14]. This is particularly true for cancer cells, which, unlike most normal cells, continually rely on glycolysis [15]. BSG acts as a chaperone to MCT1 and is necessary for its proper functioning, as BSG downregulation leads to disruption of lactate transport and decreases proliferation of tumour cells [16,17,18]. Another function of BSG that is important in cancer is its stimulatory effect on angiogenesis by inducing the expression of the pro-angiogenic vascular endothelial growth factor (VEGF) [19].

While many studies exist on BSG in solid tumours [20,21,22,23], little is known about its role in haematological malignancies. Studies on multiple myeloma (MM) proved that BSG expression is increased and this increase accompanied disease progression [24]. While both MCT1 and MCT4 were overexpressed in MM, only MCT1 downregulation led to a decrease in myeloma cell proliferation [16]. In our own study, BSG and MCT1 single nucleotide polymorphisms (SNPs) were also shown to affect MM survival [25]. In AML, BSG was shown to be co-expressed with VEGF in an immunohistochemical staining experiment, which showed it to be mostly present in the cytoplasm and cell membrane. This BSG–VEGF co-expression turned out to be a prognostic marker of overall survival in AML [26]. BSG overexpression was found to promote AML cell proliferation, and BSG inhibitor AC-73 had a potent antiproliferative effect on AML cells [27].

Apart from its membrane-bound form, BSG can also be secreted in soluble form. This can be done either by cleavage of its extracellular domain by MMPs/ADAM12, or by release of microvesicles [28,29,30]. Soluble BSG (sBSG) can be easily detected in various body fluids such as serum or plasma, and was found to be a biomarker in some diseases [31,32,33], although it was never studied in haematological malignancies. sBSG is thought to bind to membrane-bound BSG and stimulate proliferation. sBSG internalization was also shown to stimulate cells to produce more sBSG through MMP-14-mediated cleavage [34].

In the present study, we aimed to confirm the expression status of BSG and associated proteins (MCT1 and VEGF) in AML. Furthermore, we wanted to determine whether soluble BSG, as well as BSG and MCT1 genetic variants, can be used as potential markers of AML susceptibility, survival, and progression.

## 2. Materials and Methods

### 2.1. Culture of Cell Lines

Six established human AML cell lines, as well as normal human primary bone marrow CD34+ cells, were cultured in vitro for the analysis of BSG, MCT1, and VEGF expression (as previously described [35]). The normal CD34+ cells were used as a control. AML cell lines used in the study were Kasumi-3, Kasumi-1, NB4, HT93, MUTZ-3, and U937. All the cell lines were purchased from either the American Type Culture Collection (ATCC, Rockville, MD, USA) or Leibniz Institute DSMZ (Leibniz Institute DSMZ-German Collection of Microorganisms and Cell Cultures GmbH, Braunschweig, Germany) and maintained at the Cell Culture Collection of the Hirszfeld Institute of Immunology and Experimental Therapy (Wrocław, Poland). The HT93, NB4, Kasumi-1, Kasumi-3, and U937 lines were grown in RPMI-1640 (Gibco, Carlsbad, CA, USA) medium supplemented with 10% (NB4, U937) or 20% (HT-93, Kasumi-1, −3) FBS (Gibco, Carlsbad, CA, USA) and antibiotics. NB4 was additionally supplemented with GlutaMax (Gibco, Carlsbad, CA, USA). Normal CD34+ cells were grown in PromoCell Hematopoietic Progenitor Medium (PromoCell GmbH, Heidelberg, Germany) and antibiotics, while MUTZ-3 was grown in MEMalfa medium (Gibco, Carlsbad, CA, USA) supplemented with 20% FBS and 20% 5637 conditioned medium (CM). All cells were incubated with 5% CO_2_ at 37 °C. Cells and cell culture media were collected for further analyses.

### 2.2. Patients and Controls

The study involved a group of 37 newly diagnosed AML patients and 25 healthy blood donors for the analysis of serum soluble BSG, as well as a larger group of 92 newly diagnosed AML patients and 135 healthy blood donors for the analysis of BSG and MCT1 genetic variants. The healthy blood donors served as a control group. Patients in the larger group were aged 20–93, and the median age was 61. The group included 54 male and 38 female patients. The smaller group used for serum analyses was aged 25–93, and the median age was 62; there were 18 males and 19 females. Some of the patients (*n* = 27) from the smaller serum group were also included in the larger genetic group. Blood samples for all patients were obtained at diagnosis. Approval from the Wroclaw Medical University Bioethical Committee (ethical approval code: 368/2019) was obtained for the study. Clinical data analysed in the study are included in Table 1 for both groups.

### 2.3. RNA Extraction and Gene Expression Analysis

Total RNA was extracted from dry cell culture pellets using the RNeasy Plus Mini Kit (QIAGEN, Hilden, Germany) according to the manufacturer’s instructions. RNA purity and integrity were verified on the DeNovix DS-11 spectrophotometer (DeNovix Inc., Wilmington, DE, USA) and by gel electrophoresis. Using the High Capacity cDNA Reverse Transcriptase kit (Applied Biosystems, Waltham, MA, USA), 2000 ng of isolated RNA was then reverse transcribed into cDNA and an RNase Inhibitor (Applied Biosystems, Waltham, MA, USA) was added into the reaction mix. Reverse transcription was performed in a SimpliAmp Thermal Cycler (Applied Biosystems, Waltham, MA, USA) according to the manufacturer’s instructions. cDNA was then stored at −70 °C until further use.

Quantitative real-time PCR was used to evaluate BSG, MCT1, and VEGF gene expression, and the expression data were normalized to β-actin (ACTB), acting as an internal control. TaqMan Gene Expression assays and TaqMan Gene Expression Master Mix (Applied Biosystems, Waltham, MA, USA) were used for the PCR. The probes used for the reaction were: Hs00936295_m1 (BSG), Hs01560299_m1 (MCT1), Hs00900055_m1 (VEGF), and Hs01060665_g1 (ACTB). Samples were run in triplicate, and three independent experiments were performed. PCR was performed on a LightCycler 480 II (Roche Diagnostics, Rotkreuz, Switzerland) according to the manufacturer’s instructions. Relative expression was calculated using the 2^−ΔΔCt^ method.

### 2.4. ELISA Analysis

Peripheral blood was collected from 37 AML patients and 25 healthy individuals. Blood was allowed to clot and was centrifuged for 15 min at 1000× *g*. Serum was collected and stored at −70 °C until further use. Likewise, cell culture supernatants were collected (from cells cultured as described in Section 2.1), aliquoted, and stored at −70 °C. Soluble BSG and VEGF protein concentration were measured using the Human EMMPRIN/CD147 Quantikine ELISA Kit and Human VEGF Quantikine ELISA Kit (R&D Systems, Inc., Minneapolis, MN, USA) according to the manufacturer’s protocols. Absorbance was measured in a Sunrise absorbance microplate reader with Magellan analysis software (Tecan Trading AG, Männedorf, Switzerland). All samples were run in duplicate.

### 2.5. Western Blot Analysis

Western blot was used to assess the total BSG level in the lysates of cell lines. RIPA buffer with a protease inhibitor cocktail (Merck, Darmstadt, Germany) was used for cell lysate preparation. The total protein concentration of the lysates was measured using the modified Lowry method (Bio-Rad Laboratories, Inc., Hercules, CA, USA) according to the manufacturer’s instructions. Subsequently, 10–25 μg of protein samples were separated on polyacrylamide gel, and three repeats were performed, for a total of three polyacrylamide gel electrophoreses. The separated proteins were transferred to polyvinylidene difluoride (PVDF) membranes with a pore size of 0.45 μm (Merck, Darmstadt, Germany). The membranes were then incubated for 1 h with 5% non-fat dry milk in 0.1% Tris-buffered saline/Tween-20 (TBST) (Hirszfeld Institute of Immunology and Experimental Therapy, Polish Academy of Sciences, Wroclaw, Poland; Merck, Darmstadt, Germany), washed four times with TBST, and incubated overnight at 4 °C with an anti-BSG primary mouse antibody (1:200, sc-21746; Santa Cruz Biotechnology, Dallas, TX, USA). Subsequently, the membranes were washed four times with TBST, incubated for 1 h with a horseradish peroxidase (HRP)-conjugated secondary anti-mouse antibody, and then washed again four times with TBST. The Clarity Western ECL Substrate (Bio-Rad, Hercules, CA, USA) was used for chemiluminescence, and bands corresponding to BSG expression were visualized on a ChemiDoc Imaging System (Bio-Rad, Hercules, CA, USA). For β-actin detection, the membranes were first incubated for 40 min in 100% methanol to remove the remaining ECL signal, then washed four times with TBST, blocked for 1 h with 5% non-fat dry milk, and washed again four times with TBST. The membranes were then incubated with a mouse anti-β-actin-HRP monoclonal antibody (Santa Cruz Biotechnology, Dallas, TX, USA) for 1 h and washed four times with TBST. Detection was performed as described above. Densitometry was performed using ImageJ 1.48v software (U. S. National Institutes of Health, Bethesda, MD, USA) and the results were normalized to β-actin.

### 2.6. DNA Isolation, SNP Selection and Genotyping

Genomic DNA was extracted from peripheral blood taken on EDTA from 92 AML patients and 135 healthy individuals using a Qiagen DNA Isolation Kit (QIAGEN, Hilden, Germany). DNA was also isolated from AML cell lines. DNA purity and concentration were verified on the DeNovix DS-11 spectrophotometer (DeNovix Inc., Wilmington, DE, USA). Isolated DNA was subsequently stored at −20 °C until further use.

BSG and MCT1 SNPs were selected based on three criteria: (1) minor allele frequency (MAF) in European populations higher than 0.15, (2) a functional effect on expression/protein structure predicted by the National Institute of Environmental Health Sciences SNP Function Prediction tool [36], and (3) lack of high linkage disequilibrium between the SNPs. Based on these criteria, six SNPs (four in the gene coding for BSG and two in the gene coding for MCT1) were chosen. BSG and MCT1 SNP were determined using TaqMan SNP Genotyping (Applied Biosystems, Waltham, MA, USA) and LightSNiP (TIB MOLBIOL, Berlin, Germany) assays. PCR was performed on a LightCycler 480 II (Roche Diagnostics, Rotkreuz, Switzerland) according to the manufacturers’ protocols.

### 2.7. Statistical Analysis

A non-parametric Mann–Whitney U test was used for comparison of VEGF levels and clinical parameters (e.g., blasts, WBC count, haemoglobin, lactate dehydrogenase, and C-reactive protein) among AML patients with high/low sBSG. The Mann–Whitney U test and logistical regression were used for comparison of sBSG levels between patients and healthy individuals. A one-way ANOVA with Dunnett’s multiple comparison test and multiplicity adjusted *p*-value was used to compare differences between BSG/MCT1/VEGF expression/sBSG/total BSG level in AML cell lines vs. normal CD34+ cells. Survival was analysed using the Kaplan–Meier method, while Spearman’s coefficient was used to assess the correlation between sBSG and clinical parameters and VEGF. The Mann–Whitney U test, Kaplan–Meier survival analysis, and Spearman’s correlation analysis were performed in a Real Statistics Resource Pack for Microsoft Excel 2013 (version 15.0.5023.1000, Microsoft, Redmont, WA, USA), logistical regression was performed in RStudio (RStudio, PBC., Boston, Massachusetts, USA), and the ANOVA with Dunnett’s test was performed in GraphPad Prism (version 8.0.1, GraphPad Software, San Diego, CA, USA). Linkage disequilibrium and Hardy–Weinberg equilibrium analyses were performed with the Haploview 4.2 software. Fisher’s exact test was used to test if allele/genotype frequencies differed between patients and controls, as well as between subgroups of patients. This was calculated using the web-based tool http://vassarstats.net/tab2x2.html (accessed on 29 September 2021). *p*-values < 0.050 were considered statistically significant, while those between 0.050 and 0.100 were considered to be indicative of a trend.

## 3. Results

### 3.1. Expression of BSG, MCT1 and VEGF mRNA in AML and Control Cell Lines

**Figure 1:**
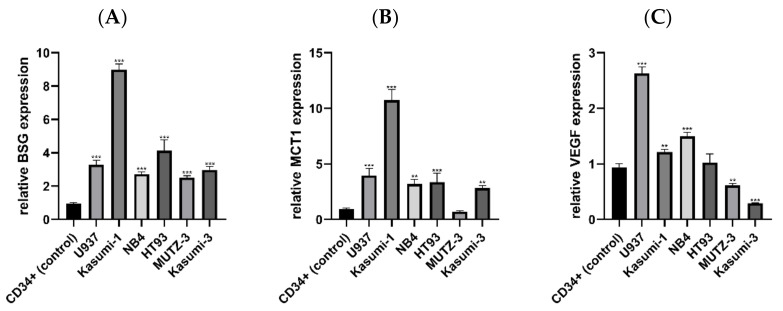
Relative expression of BSG (**A**), MCT1 (**B**), and VEGF (**C**) mRNA in acute myeloid leukaemia (AML) cell lines and normal primary bone marrow CD34+ cells. Expression of BSG is uniformly higher in all AML lines, while MCT1 is overexpressed in all lines except for MUTZ-3. Expression of VEGF is not consistent, although it is either higher than or similar to normal CD34+ cells in most AML lines. Each bar represents results from three independent experiments. The significance of the differences between individual lines and the normal CD34+ cells is indicated as: *p* < 0.050, ** *p* < 0.010, *** *p* < 0.001.

To assess the general BSG expression level in AML, we analysed BSG mRNA levels in six AML cell lines (U937, Kasumi-1, NB4, HT93, MUTZ-3, and Kasumi-3), as well as in normal primary bone marrow CD34+ cells. Relative BSG expression was significantly higher in all AML lines compared to the normal CD34+ cells (*p* < 0.001; Figure 1A). Expression was the highest in Kasumi-1 (8.99 relative to CD34+ cells), and the lowest in MUTZ-3 (2.51 relative to normal CD34+ cells), while the median expression level of all AML lines was 3.14.

Additionally, we assessed the mRNA expression of MCT1 and VEGF, the two proteins associated with BSG function in cancer. MCT1 expression was higher in all AML lines excluding MUTZ-3 (*p* < 0.010), which had slightly lower MCT1 expression compared to normal CD34+ cells (0.70 relative to normal CD34+ cells; Figure 1B). As with BSG, Kasumi-1 was characterized by the highest level of MCT1 expression (10.76 relative to CD34+ cells), while the median expression level of all AML lines (including MUTZ-3) was 3.36. Unlike MCT1 and BSG, relative VEGF expression was not uniform among AML lines, although most AML lines had expression levels higher than or similar to normal CD34+ cells (Figure 1C). The highest expression was observed in U937 cells (2.63 relative to normal CD34+ cells). Two lines (Kasumi-1, NB4) had expression slightly higher than normal CD34+ cells. One was not significantly different from the control (HT93), while two lines (Kasumi-3 and MUTZ-3) had lower expression than the control.

### 3.2. Soluble BSG in Serum as a Marker of AML Susceptibility and Survival

**Figure 2:**
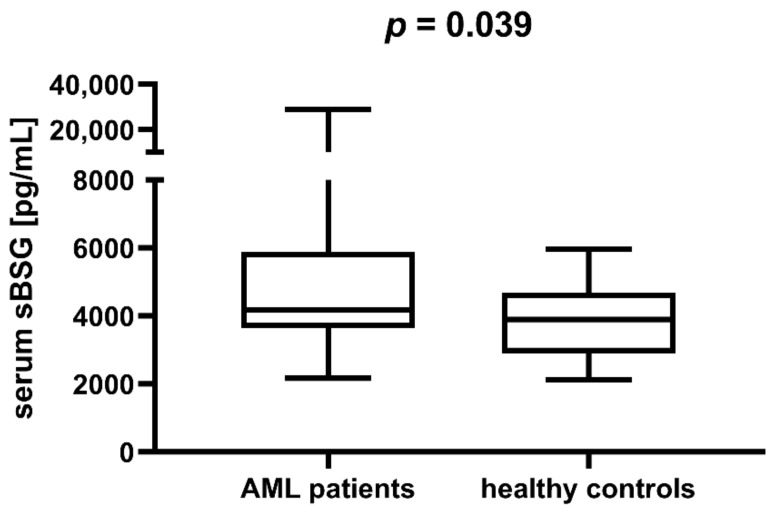
Serum soluble basigin (sBSG) in acute myeloid leukaemia (AML) patients is higher than in healthy individuals.

Having proven the increased expression of BSG in AML, we attempted to analyse the expression of soluble BSG (sBSG) in the serum of AML patients (*n* = 37) and healthy individuals (*n* = 25). The median and interquartile range were 4186.45 pg/mL (IQR = 3658.70–5710.65) for the former and 3894.45 pg/mL (IQR = 2903.50–4544.15) for the latter group. Comparing sBSG levels in the two groups, we found that sBSG was higher in the serum of AML patients compared to healthy individuals (*p* = 0.039, Figure 2). Additionally, we performed an age- and sex-adjusted logistical regression analysis that included the level of sBSG as a potential factor differentiating cases from controls. This analysis seems to confirm the trend of higher sBSG in AML patients (*p* = 0.077). This indicates that serum sBSG may be a potential marker of AML.

**Figure 3:**
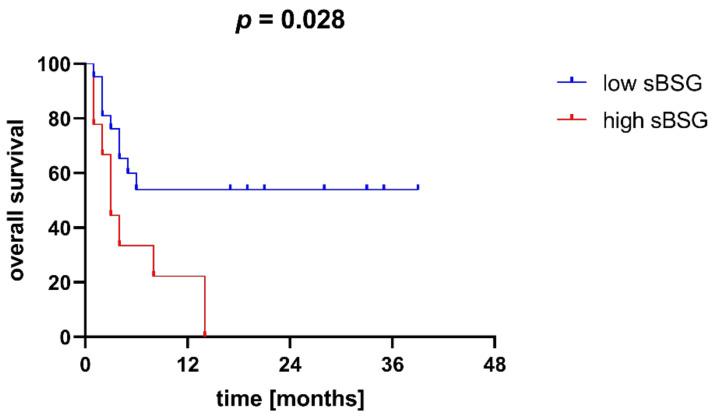
Overall survival (OS) of patients with high and low soluble BSG (sBSG) serum levels. Patients with high sBSG are characterized by significantly worse OS. The cut-off for high BSG was the upper quartile among the studied patients, or 5710.65 pg/mL.

For further analyses, we divided our cohort of AML patients into two groups according to their sBSG level—those with high sBSG (values above the upper quartile, or 5710.65 pg/mL) and those with low sBSG (values below the upper quartile). Using the Kaplan–Meier method, we found that patients in the high sBSG group had significantly worse overall survival (OS) than patients in the low sBSG group (*p* = 0.028; Figure 3).

**Figure 4:**
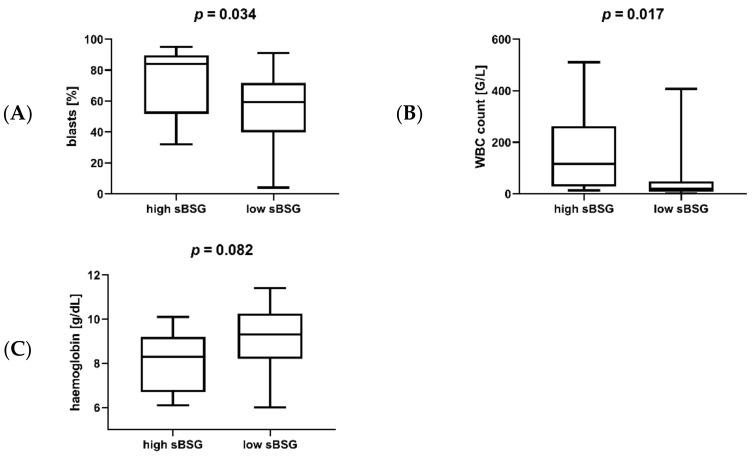
Percentage of blasts in the bone marrow (**A**), white blood cell (WBC) count (**B**), and haemoglobin levels (**C**) in AML patients with high and low sBSG. Patients with high sBSG were characterized by a higher percentage of blasts, higher WBC count, and lower haemoglobin levels, although the latter association was not statistically significant. Increased percentage of blasts, increased WBC count, and decreased haemoglobin are all hallmarks of more advanced AML. The cut-off for high sBSG was the upper quartile among the studied patients, or 5710.65 pg/mL.

In the next step, we compared sBSG with various clinical parameters of AML. Soluble BSG was positively correlated with the percentage of blasts in the bone marrow (*p* = 0.017, R = 0.441), and negatively with haemoglobin (*p* = 0.049891, R = −0.339), and a trend for a positive correlation with white blood cell (WBC) count was observed (*p* = 0.061, R = 0.325). In terms of groups with different (high/low, as defined in the previous paragraph) sBSG expression, patients with high sBSG were characterized by a higher percentage of bone marrow blasts (*p* = 0.034; Figure 4A) and higher white blood cell count (*p* = 0.017; Figure 4B) compared to low sBSG patients. A trend for lower haemoglobin (*p* = 0.082; Figure 4C) was also observed. However, sBSG did not correlate with the serum levels of the angiogenic factor VEGF (*p* = 0.786, R = 0.048).

**Figure 5:**
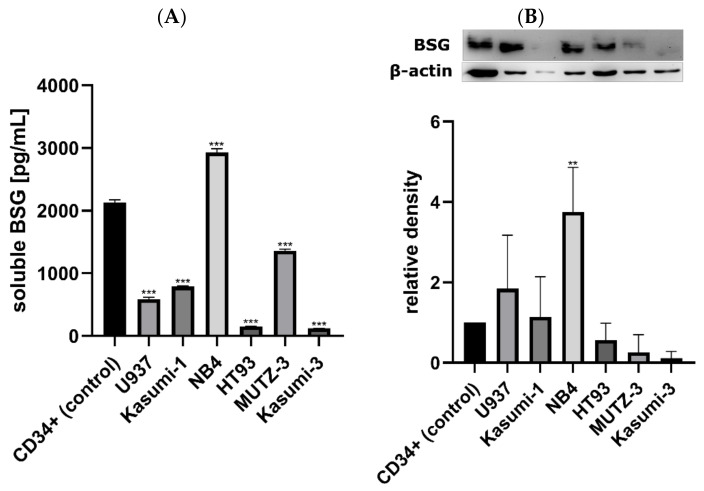
Protein expression of BSG in AML cell lines and normal CD34+ cells. (**A**) shows secreted soluble BSG in cell culture supernatants, while (**B**) shows the total BSG levels in cell lysates of AML cell lines and normal CD34+ cells normalized to β-actin. Protein expression of BSG in relation to normal CD34+ cells differs between various AML lines and is not consistent, although most cell lines exhibit lower soluble BSG levels than the control (**A**), and total BSG levels higher than or similar to the control (**B**). Each bar represents results from three technical repeats, and representative immunoblots for BSG and β-actin are shown above the plot (**B**). The significance of the differences between individual lines and the normal CD34+ cells is indicated as: *p* < 0.050, ** *p* < 0.010, *** *p* < 0.001.

Soluble BSG was also measured in supernatants of AML cell lines and normal CD34+ cells. All AML lines except for NB4 (2928.93 pg/mL) secreted lower sBSG levels than normal CD34+ cells (2129.42 pg/mL; Figure 5A). The median sBSG level for all AML lines was 687.06 pg/mL and two lines (Kasumi-3 and HT93) secreted sBSG at barely detectable levels (120.98 and 152.22 pg/mL, respectively). Similarly, we measured the total BSG in AML cells and normal CD34+ cells. Three cell lines (U937, Kasumi-1, and NB4) had higher BSG levels compared to normal CD34+ cells (corresponding to higher levels of their mRNA expression), but one had similar levels (HT93), and two had lower levels (MUTZ-3, Kasumi-3; Figure 5B). Interestingly, the total BSG was strongly correlated with VEGF mRNA expression in AML cell lines (*p* = 0.005, R = 0.943).

### 3.3. BSG and MCT1 Genetic Variants Are Associated with Survival and Other Clinical Parameters of AML

We genotyped a group of AML patients (*n* = 92) and healthy individuals (*n* = 135) for four BSG (rs4919859, rs4682, rs8637, and rs8259) and two MCT1 (rs1049434 and rs9429505) SNPs. The frequencies for all the SNPs were in accordance with the Hardy–Weinberg equilibrium in both groups. No statistically significant differences between patients and healthy individuals were detected, suggesting no association of selected SNPs with the predisposition to AML. The distribution of genotypes is presented in Table 2.

**Figure 6:**
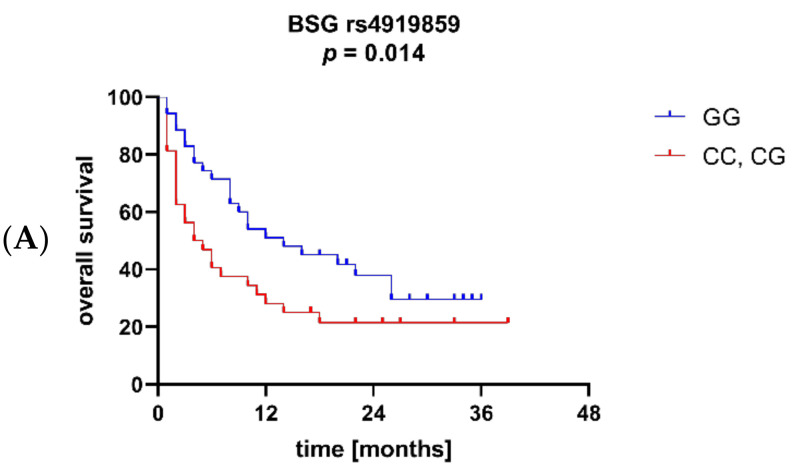

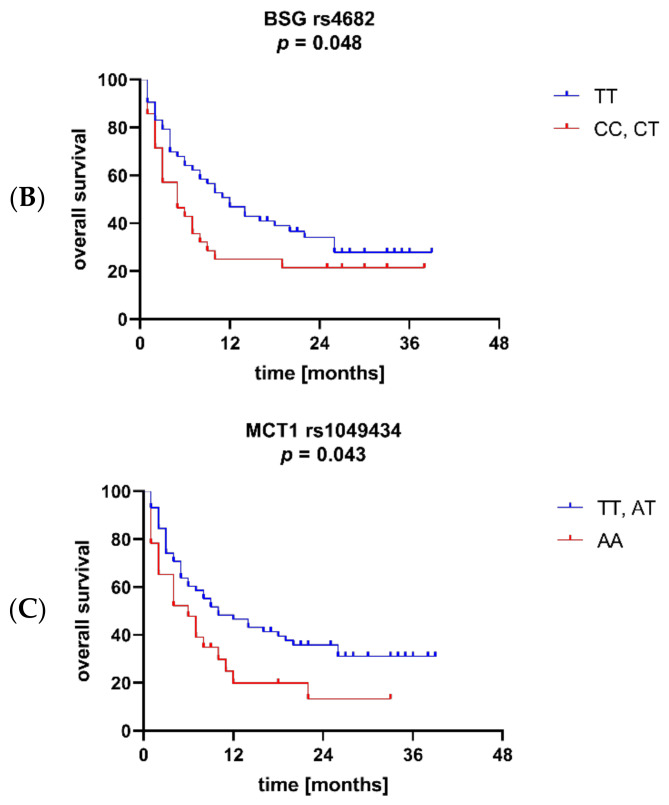
Overall survival (OS) in acute myeloid leukaemia (AML) patients with variants of basigin (BSG) and monocarboxylate transporter 1 (MCT1) SNPs. Patients with BSG alleles rs4919859 C (**A**) and 4682 C (**B**) were characterized by worse OS, while those with MCT1 allele rs1049434 T (**C**) had better OS.

The Kaplan–Meier method was used to assess the effects of BSG and MCT1 SNPs on overall survival. The analysis showed that BSG alleles rs4919859 C and rs4682 C were associated with significantly worse OS (*p* = 0.014 and *p* = 0.048, respectively; Figure 6A and 6B), while carriers of MCT1 allele rs1049434 T were characterized by better OS (*p* = 0.043; Figure 6C). Additionally, a trend towards better OS in patients with MCT1 allele rs9429505 A was observed (*p* = 0.063). No association between the SNPs and soluble BSG levels was found in AML patients.

We also analysed BSG and MCT1 SNPs in relation to various clinical parameters of AML. We observed that BSG alleles rs4682 C and rs8259 A were more common in AML subtypes M0–M2 (myeloblastic leukaemia with/without maturation) than in other subtypes (*p* = 0.017 and *p* = 0.006, respectively). Additionally, MCT1 allele rs9429505 G was associated with higher CRP (*p* = 0.049).

## 4. Discussion

BSG (CD147) is a multifunctional glycoprotein involved in several regulatory pathways associated with cell adhesion, migration, proliferation, angiogenesis, ion transport, or drug efflux. Recent findings on its potential involvement in SARS Coronavirus 2 (SARS-CoV-2) infection increased the need to better describe BSG expression patterns in various tissues [6,7]. BSG was studied in many solid tumours, as well as recently in multiple myeloma and acute lymphoblastic leukaemia [20,21,22,23,24,25,37,38,39,40]. However, relatively little is known about its involvement in acute myeloid leukaemia (AML).

In the present study, we aimed to clarify the expression status of BSG and MCT1 RNA in AML cells and to study soluble serum BSG as well as BSG and MCT1 genetic variants as potential biomarkers of AML risk, survival, and progression. We found BSG and MCT1 mRNA expression to be significantly increased in almost all AML cell lines compared to normal primary bone marrow CD34+ cells. This confirms earlier reports regarding potential BSG and MCT1 overexpression in AML [26,27,41]. This pattern of BSG and MCT1 overexpression was also observed earlier in multiple myeloma [16,42]. Unusually, MCT1 showed decreased expression in one cell line, MUTZ-3, which is a model for M4 AML, or acute myelomonocytic leukaemia. The reason for this is unclear and requires further study. Nonetheless, the BSG level was still increased in this cell line.

It is established that angiogenesis plays a role in AML [43]. Since BSG was previously shown to stimulate expression of the pro-angiogenic factor VEGF [19], and VEGF was shown to be co-expressed with BSG in bone marrow samples from AML patients [26], we decided to assess VEGF expression in AML cell lines. However, the pattern of VEGF mRNA expression differed from that of BSG: some lines exhibited overexpression (NB4, U937, Kasumi-1), some had similar expression levels to the normal cells (HT93), while others had decreased expression (Kasumi-3, MUTZ-3). However, VEGF was strongly correlated with the total protein BSG, as measured by western blot in AML cells. This seems to be in line with the previously proposed mechanism of VEGF upregulation through membrane-bound BSG and MMPs, and may confirm the importance of BSG in VEGF regulation in AML cells.

A major goal of our study was to determine whether BSG is a potential biomarker in AML. While it works primarily as a membrane-bound protein, various studies have found soluble circulating BSG to be a marker of disease [31,32,33]. In this study, we showed for the first time that sBSG is more abundant in the serum of AML patients than healthy individuals. Moreover, patients with high sBSG were found to have worse overall survival, more blasts in the bone marrow, higher WBC count, and lower haemoglobin levels. All of this suggests that sBSG may be a marker of worse survival, as a high percentage of blasts and high WBC count are major hallmarks of AML. Additionally, patients often have decreased haemoglobin due to anaemia resulting from impaired bone marrow function. The mode of action of sBSG is not fully understood, but it is believed that it can dimerize with membrane-bound BSG to exert its influence [34,44]. Interestingly, we did not observe increased sBSG in AML cell culture supernatants, even though the cells exhibited increased levels of BSG mRNA. Actual protein production may not necessarily correspond to mRNA levels, and our results show that not all of the cell lines produced total BSG in levels corresponding to their BSG mRNA expression. However, this does not seem to explain the decreased sBSG secretion in almost all of the investigated cell lines. Furthermore, an earlier study suggests that total protein production of BSG does correlate with mRNA expression in AML cells [27]. A significant factor influencing the observed levels of sBSG may be the way in which sBSG is secreted. BSG is primarily expressed on the cell surface, and only some of its molecules are released, either by MMPs/ADAM12-mediated cleavage, or in microvesicles [28,29,30]. This means that sBSG levels may be regulated by more factors and do not only reflect mRNA and protein BSG expression. Furthermore, sBSG may be released by other cells from the bone marrow microenvironment, following stimulation by AML cells. A study on gingival fibroblasts and U937 cells found that their co-culture released more sBSG than the respective cultures did separately [45]. The same study also confirmed that sBSG participated in a self-regulatory mechanism with its membrane-bound form [45]. An earlier study also suggested that sBSG internalization induced secretion of more sBSG [34], acting in a positive feedback loop. All this evidence suggests that sBSG is likely not causative in the associations with AML described here, but it can still be considered a potentially useful diagnostic and prognostic biomarker.

Another aspect of this study was to analyse SNPs in the gene coding for BSG and MCT1 in the context of AML. We previously showed that SNPs can act as biomarkers in AML [46,47], and we identified some BSG and MCT1 variants that were associated with survival in multiple myeloma patients; most importantly, BSG allele rs4919859 C was associated with worse MM progression-free survival [25]. In the current study, allele rs4919859 C was likewise associated with adverse overall survival in AML patients. However, no association was observed in AML patients for BSG allele rs8637 G, which conferred worse survival on MM patients [25]. The rs4919859 BSG SNP is predicted to lie in a transcription factor binding site [35], and therefore is expected to exert its influence by affecting BSG transcription rates. Indeed, we observed that AML cell lines with the highest BSG RNA expression (Kasumi-1 and HT-93) were homozygous for BSG rs4919859 C. Nevertheless, this SNP remains relatively under-studied; other than the current study and our previous work on MM patients [25], only one study has analysed it in the context of coronary heart disease [48]. Another SNP found to be associated with AML survival in the current study is rs4682, which is predicted to be located in an exonic splicing enhancer/silencer (ESE/ESS) [36].

We also analysed two MCT1 SNPs, one of which, rs1049434 (allele T), was associated with better overall survival. This SNP represents a missense Asp (A) to Glu (T) mutation, and the Glu (T) isoform is thought to be better at transporting lactate [49]. The effect of this SNP seems to be inconsistent between different diseases and conditions [25,50,51,52]. This may be due to MCT1’s being quite elastic in its role as a transporter, as it can transport monocarboxylates bidirectionally [13]. In the case of AML, the effect of rs1049434 on survival may be influenced by the fact that MCT1 was recently found to be more important in lactate uptake, rather than efflux, in differentiating AML cells [42]. This is due to the fact that AML cells, unlike most cancer cells, often employ oxidative phosphorylation for energy production [53]. The dual nature of MCT1 may be the reason behind the varying effects of the rs1049434 SNP between our work and previous works, although this remains to be confirmed in a separate study.

## 5. Conclusions

In conclusion, our study shows that BSG and MCT1 are overexpressed in most AML cells compared to normal primary bone marrow CD34+ cells. Soluble serum BSG is increased in AML patients compared to healthy individuals and its high level is associated with lower overall survival and worse clinical parameters. BSG and MCT1 genetic variants are associated with overall survival and other clinical parameters. This study confirms the role of BSG and MCT1 in AML and shows that soluble serum BSG and BSG/MCT1 genetic variants may act as markers of AML survival.

## Figures and Tables

**Table 1:** Characteristics of AML patients included in the study.

Data	Serum Study Group Range and Median (*n* = 37) ^1^	Genetic Study Group Range and Median (*n* = 92) ^1^
age	25–93, med = 62	20–93, med = 61
bone marrow blasts (%)	4–95, med = 68	20–98, med = 65
white blood cell count (G/L)	0.8–510.5, med = 22.7	0.7–510.5, med = 21
haemoglobin (g/dL)	6.0–11.4, med = 8.8	6.1–19.5, med = 9.2
platelets (G/L)	5–393, med = 44.5	2–460, med = 52
lactate dehydrogenase (U/L)	168–2170, med = 446.5	26–2535, med = 369
C-reactive protein (mg/L)	2.3–115.4, med = 36.3	1.1–359, med = 25

^1^ The serum study group (*n* = 37) was used for analysis of serum soluble BSG, while the larger genetic study group (*n* = 92) was used for analyses of BSG and MCT1 genetic polymorphism.

**Table 2:** Distribution of basigin (BSG) and monocarboxylate transporter 1 (MCT1) genotypes in acute myeloid leukaemia (AML) patients and healthy individuals.

	AML Patients	Healthy Individuals
BSG rs4919859		
CC	18 (19.6%)	19 (14.1%)
CG	35 (38.0%)	54 (40.0%)
GG	39 (43.4%)	62 (45.9%)
BSG rs4682		
CC	7 (7.6%)	4 (3.0%)
CT	26 (28.3%)	35 (25.9%)
TT	59 (64.1%)	96 (71.1%)
BSG rs8637		
AA	30 (32.6%)	47 (34.8%)
AG	45 (48.9%)	61 (45.2%)
GG	17 (18.5%)	27 (20.0%)
BSG rs8259		
TT	50 (54.3%)	78 (57.8%)
TA	31 (33.7%)	49 (36.3%)
AA	11 (12.0%)	8 (5.9%)
MCT1 rs1049434		
AA	28 (30.4%)	54 (40.0%)
AT	45 (48.9%)	58 (43.0%)
TT	19 (20.7%)	23 (17.0%)
MCT1 rs9429505		
AA	52 (56.5%)	67 (49.6%)
AG	34 (37.0%)	60 (44.4%)
GG	6 (6.5%)	8 (5.9%)

## Data Availability

The data presented in this study are available upon request from the corresponding author. The data are not publicly available due to privacy or ethical restrictions.
